# Transforming Growth Factor-β Drives Epithelial Mesenchymal Transition and Reduces Synthesis of Unelaborated O-GalNAc Glycans in Breast Cancer Cells

**DOI:** 10.1369/00221554261416994

**Published:** 2026-02-28

**Authors:** Joanna Cull, Ryan C. Pink, Priya Samuel, Susan A. Brooks

**Affiliations:** School of Biological & Medical Sciences, Oxford Brookes University, Oxford, UK (JC, RCP, PS, SAB)

**Keywords:** EMT, epithelial-mesenchymal transition, *Helix pomatia* agglutinin, O-glycosylation, O-GalNAc glycans, TGF-β1, Tn antigen, transforming growth factor beta-1

## Abstract

Altered O-glycosylation of cancer cells is frequently associated with metastasis and poor prognosis. During metastasis, cells must also lose their epithelial characteristics and become mesenchymal and motile, termed epithelial-mesenchymal transition (EMT). While it is established that transforming growth factor beta-1 (TGF-β1) can induce EMT, the effect of cytokine-induced EMT on O-glycosylation of breast cancer cells has not previously been explored. MCF-7 and T47-D breast cancer cells were treated with TGF-β1 over a time course of up to 7 days. Morphological changes were assessed using confocal microscopy and quantified; levels of EMT marker expression were assessed using immunofluorescence with confocal microscopy and western blot. The effect of TGF-β1 on synthesis of unelaborated Tn antigen was explored using *Helix pomatia* agglutinin (HPA) labelling. TGF-β1 treatment induced morphological changes, resulting in an elongated, mesenchymal-like phenotype, and a reduction of epithelial marker E-cadherin but did not detectably induce mesenchymal markers N-cadherin or vimentin. It also resulted in a significant reduction in unelaborated Tn antigen, detected by HPA labelling. These observations are consistent with TGF-β1 inducing an ‘early’ or partial EMT state over this timeframe, and a concomitant change in O-glycosylation, consistent with the synthesis of more elaborated O-glycan structures; such glycoplasticity may function in metastasis. **(J Histochem Cytochem XX:XXX–XXX, XXXX)**

## Introduction

**Figure 1.**
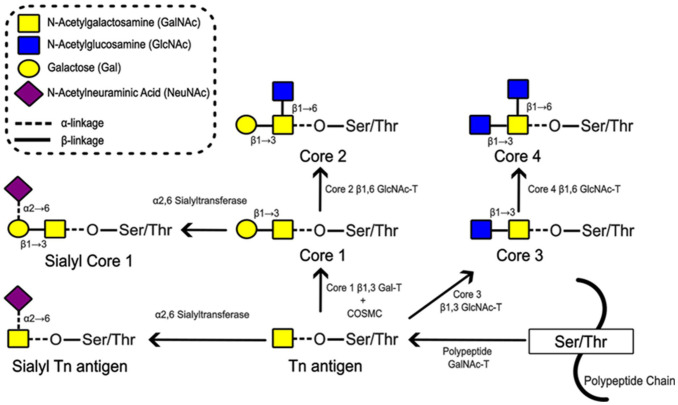
Initial steps in O-linked glycosylation and synthesis of core structures 1 to 4. Mucin-type O-glycosylation is initiated by the addition of an N-acetylgalactosamine (GalNAc) monosaccharide onto a serine (Ser) or threonine (Thr) of a protein to form the Thomsen nouvelle or Tn antigen (GalNAcα1-O-Ser/Thr). In healthy adult cells, Tn antigen is always further elaborated. Cores 1 to 4 are the commonest core structures to be synthesised. Core 1 (also called Thomsen-Friedenreich or T antigen), Galβ1-3GalNAcα1-O-Ser/Thr, is formed by addition of a β1,3 Gal to the Tn antigen. It can be elaborated to form core 2 by the addition of a β1,6-linked N-acetylglucosamine (GlcNAc). Core 3 is formed by addition of a β1,3 GlcNAc to Tn antigen, and may be extended by the addition of a β1-6 GlcNAc to form core 4. The core structures are then usually extended by the addition of further, stepwise addition of monosaccharides to form more complex glycans. Both Tn antigen and core 1 may be sialylated to form sialyl Tn or sialyl core 1, respectively, and this prevents their further chain extension. Monosaccharide symbols follow the SNFG (Symbol Nomenclature for Glycans) system,^1^ details of which can be found at NCBI (https://www.ncbi.nlm.nih.gov/glycans/snfg.html).

It is well-established that changes in glycosylation are a common feature of cancers and may have functional and prognostic significance. A frequently reported alteration is incomplete synthesis or truncation of O-linked glycans, including the exposure of the Tn antigen (GalNAcα1-O-Ser/Thr) which, due to chain extension, is normally cryptic in healthy adult tissues. The initiation and early steps in O-glycan synthesis, with their resulting glycan motifs, are illustrated in Fig. 1 for reference (monosaccharide symbols follow the SNFG, Symbol Nomenclature for Glycans, system).^1^ The presence of Tn antigen was first described in human cancers by Prokop and Uhlenbruck in 1969,^2^ and as many as 90% of cancers of epithelial origin synthesise unelaborated Tn antigen, including carcinomas of the breast, lung, bladder, cervix, ovary, stomach and prostate.^3,4^ Recently, a survey of more than 700 cancer cases reported high levels of Tn antigen in breast, colorectal and pancreatic cancer and negligible levels in corresponding normal tissue.^5^ The presence of Tn antigen can be revealed through the binding of the lectin *Helix pomatia* agglutinin (HPA) which recognises terminal α-GalNAc residues. Leathem and Brooks^6^ were first to report that the presence of HPA-binding glycans in primary breast cancer was associated with poor long-term patient prognosis, aggressive biological behaviour and metastasis. Many other studies have since confirmed that HPA labelling is associated with lymph node and distant metastases, and poorer disease-free and overall survival in breast – and other – cancers (see Brooks et al.^7–9^ for review). Exposure of other truncated O-glycans – including core 1 (Galβ1-3GalNAcα1-O-Ser/Thr, also known as Thomsen-Friedenreich or T antigen), sialyl Tn (Neu5Acα2-6GalNAcα1-O-Ser/Thr), and sialyl core 1 (Neu5Acα2-3Galβ1-3GalNAcα1-O-Ser/Thr), are also commonly reported in a range of cancers, and their presence also frequently associated with aggressive biology and poor prognosis (reviewed by Brooks et al.,^9^ Qusairy and Rada^10^).

Epithelial-mesenchymal transition (EMT) is a biological process whereby basement membrane-bound, polarised, adhesive epithelial cells transdifferentiate into migratory, invasive mesenchymal cells. This requires changes in cellular architecture, morphology, adhesion and migratory capabilities and is critical to cancer metastasis.^11^ It is a highly plastic, reversible process, and mesenchymal-epithelial transition (MET) allows for the transition of motile, mesenchymal cells back into polarised epithelial cells.^12^ This plasticity features throughout the metastatic cascade: epithelial cancer cells in the primary tumour must become mesenchymal and motile in order to detach and invade locally and into blood vessels and lymphatics; then transdifferentiate into an epithelial phenotype in order to adhere to the endothelium; subsequently they become mesenchymal during extravasation and invasion at a secondary site; and finally return to an epithelial phenotype to establish a secondary tumour.^13^ There is mounting evidence of a relationship between EMT and O-glycosylation, such that the family of glycosyltransferases that initiate mucin-type O-linked glycosylation, the UDP-N-acetyl-α-D-galactosamine:polypeptideN-acetylgalactosaminyltransferases (ppGalNAc-Ts, GalNAc-Ts), coded for by the GALNT gene family, may be considered to be the ‘master regulators’ of EMT.^13^

A variety of cytokines have been implicated in the induction of EMT, including epidermal growth factor (EGF), fibroblast growth factor (FGF), hepatocyte growth factor (HGF), insulin-like growth factor (IGF) 1 and 2 and transforming growth factor beta (TGF-β).^14^ Other cytokines secreted by the tumour stroma can act to promote EMT, including interleukin-6 (IL-6)^15^, interleukin-8 (IL-8)^16^ and tumour necrosis factor alpha (TNF-α).^17^ Cytokines bind to their respective receptors and induce a range of signalling pathways. The pathways of EMT-inducing cytokines converge and result in the regulation of many EMT-inducing transcription factors, including Snail, Slug, TWIST1/2 and ZEB1/2, which act to suppress the transcription of E-cadherin and induce transcription of N-cadherin, vimentin and fibronectin, promoting cell polarity loss and destroying tight junctions.^18,19^ Incomplete activation of signalling pathways and transcription factors can result in a partial EMT state.^20^

The pleiotropic cytokine TGF-β has received much attention due to its efficiency in inducing EMT and its strong link to EMT induction in both normal development and disease states.^21,22^ The role of TGF-β in inducing EMT was first recognised by Miettinen et al.^23^ in cell culture of mouse mammary epithelial cells, through observation of changes in morphology (to elongated, spindle-shaped morphology), decreased expression of E-cadherin and increased fibronectin, detected by western blotting and immunofluorescence. It is now well-accepted that breast epithelial cell cultures can be treated with TGF-β to reliably induce EMT (reviewed in Derynck and Weinberg),^24^ including in MCF-7^25–29^ and T-47D^26^ breast cancer cell lines.

In humans, the TGF-β superfamily of cytokines consists of 33 members, which include three TGF-β isoforms in the TGF-β subfamily (TGF-β1, TGF-β2 and TGF-β3), bone morphogenetic proteins (BMPs) and growth differentiation factors (GDFs).^30^ An interesting duality exists where TGF-β acts as both a tumour suppressor and tumour promoter during various stages of cancer development and metastasis. This has been referred to as the ‘TGF-β paradox’.^31,32^ TGF-β inhibits proliferation and can induce apoptosis of premalignant cells under normal physiological conditions. However, in the later stages of malignancy, TGF-β can induce EMT and facilitate cell migration and invasion (reviewed by Bierie and Moses).^33^ This switch in late-stage cancer is thought to be due to genetic, epigenetic and tumour microenvironment changes.^31^ TGF-β1 is the most abundant, prototypical isoform of the TGF-β family. It is expressed ubiquitously in human tissues and is typically upregulated to a greater degree than the other isoforms in cancer.^33,34^ Literature investigating changes in O-glycosylation following TGF-β treatment in the context of cancer metastasis is very limited. In this study, to elucidate whether glycosylation changes occur in a cytokine-induced EMT state, we examined the effect of TGF-β1 treatment on MCF-7 and T-47D breast cancer cell lines.

## Materials and Methods

### Cell Culture

Cell lines were originally obtained from the European Collection of Authenticated Cell Cultures (ECACC, Salisbury, UK). MCF-7 cells were routinely cultured in Dulbecco’s modified Eagle medium (DMEM)/ F12/ GlutMAX (11514436, Gibco/Thermo Fisher, Loughborough, UK) with 10% v/v heat inactivated foetal calf serum (FCS) (11550356, Gibco/Thermo Fisher, Loughborough, UK) and T-47D cells were cultured in Roswell Park Memorial Institute (RPMI) medium-1640 with L-glutamine (10-040-CV, Corning, Amsterdam, The Netherlands) and 10% v/v heat inactivated FCS. Cells were cultured in a humidified atmosphere at 37C with 5% v/v CO₂ and were negative for mycoplasma contamination. Cells were cultured for a maximum of 20 passages before being discarded.

To culture cells on coverslips for subsequent microscopy, 13mm diameter glass coverslips were sterilised in industrial methylated spirit, transferred into the wells of a 24-well cell culture plate and allowed to air dry. Cells at around 70% confluency was dissociated from culture flasks using trypsin/ethylenediaminetetraacetic acid (EDTA) (15400054, Gibco/Thermo Fisher, Loughborough, UK). A suspension of cells (MCF-7 at 8000 cells/ml and T-47D at 18,000 cells/ml) was created in their respective preferred complete growth medium. 1ml of cell suspension was added into each well of the plate, the plate was swirled once and then incubated at 37C and 5% v/v CO2 under humidified conditions for 72 hr to allow cells to adhere to the coverslips and grow to around 70% confluency.

### TGF-β1 Treatment of Cells

**Figure A1.**
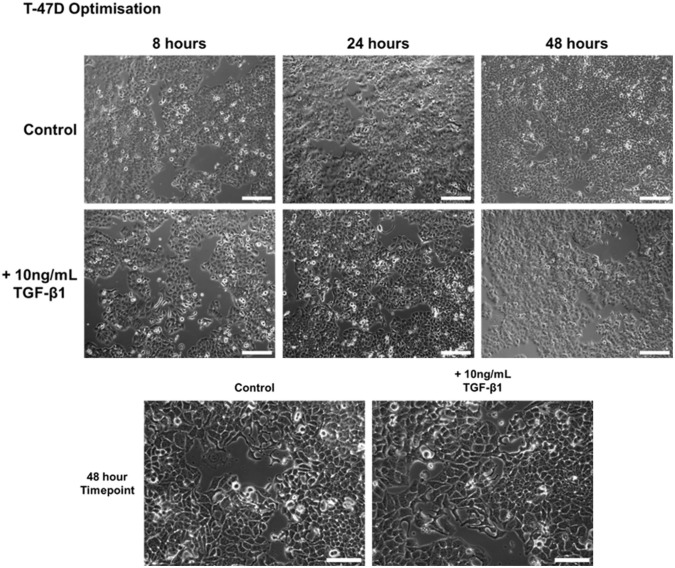
T-47D cell line transforming growth factor-β1 (TGF-β1) treatment optimisation – morphology assessment. Visual morphology inspections showed no visually discernible changes up to the 48-hr treatment timepoint. Scale bar = 50µm.

**Figure A2.**
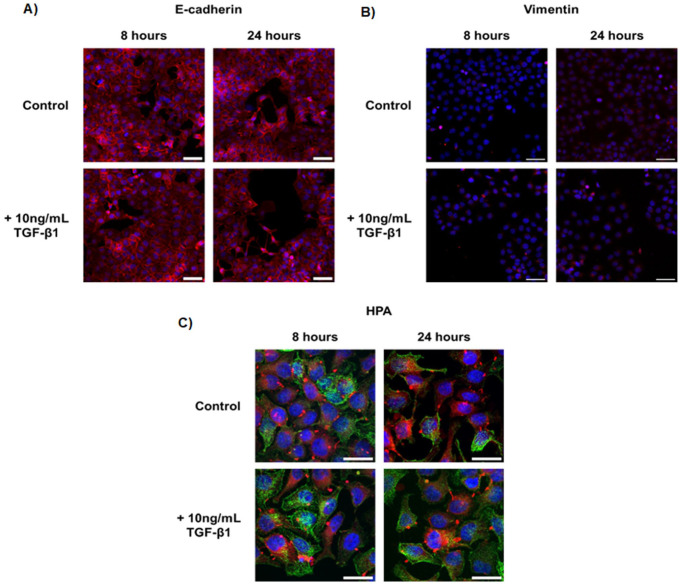
T-47D cell line TGF-β1 treatment optimisation – E-cadherin, vimentin and *Helix pomatia agglutinin* (HPA) assessment. Visual inspections of E-cadherin, vimentin and HPA labelling showed no visually discernible changes up to the 8-hr and 24-hr treatment timepoints. (A) E-cadherin (red) and DAPI nuclear stain (blue). Scale bar = 50µm. (B) Vimentin (red) and DAPI nuclear stain (blue). Scale bar = 50µm. (C) HPA (green) for Tn antigen, CellMask for plasma membrane labelling (red) and DAPI nuclear stain (blue). Scale bar = 25µm.

Complete cell culture media was aspirated from culture plates and replaced with media containing low (1% v/v) FCS for 24 hr. Following this, low FCS cell culture media containing 10ng/ml TGF-β1 (100-21C, Thermo Fisher, Loughborough, UK) was added and replenished every other day. Control cells were cultured in low FCS cell culture media and replenished at the same time as those subject to treatment conditions. Timepoints of 24, 48, 72 hr and 7 days post initial TGF-β1 treatment were analysed for MCF-7 cells. Timepoints of 72 hr and 7 days post initial TGF-β1 treatments were analysed for T-47D cells, since the T-47D cell line demonstrated no visually discernible morphological changes at the 8, 24 or 48-hr timepoint or change in EMT markers E-cadherin or vimentin at the 8 and 24-hr timepoint (see Appendix Figs. A1 and A2).

### Analysis of Cell Morphology Following TGF-β1 Treatment

Images of cells were captured under phase contrast using a Zeiss Primovert inverted microscope with AxioCam IC (Carl Zeiss, Oberkochen, Germany) and a Primo Plan-ACHROMAT 10x/0.25 Ph1 objective. Morphological analysis of cells was conducted using the freehand tool in FIJI (NIH, Bethesda, MD) to create regions of interest (ROIs) on up to 300 cells, or every cell if the total was less than 300, in an image. This was performed across three images per technical replicate, each technical replicate included analysis of a minimum of 608 cells, and three biological replicates were conducted. Measurements were captured of the aspect ratio (length/width) and the circularity (4π*(area)/(perimeter)^2^) of cells. The major (x) and minor (y) aspects of a cell dictate the aspect ratio (x:y ratio), and a value of >2:1 was classified as an ‘elongated’ morphology and any value <2:1 was classified as having a ‘rounded’ morphology. For circularity, a value of 1.0 indicates a perfect circle and values <1.0 indicate an increasingly elongated shape.

### Western Blotting for EMT Markers E-Cadherin, Vimentin and N-Cadherin in Cells Following TGF-β1 Treatment

For western blotting, cells were seeded into 6-well cell culture plates (MCF-7 at 10,000 cells/ml and T-47D at 18,000 cells/ml). Standard cell culture media was aspirated and replaced with low FCS cell culture media for 24 hr. Following this, low FCS cell culture media containing 10ng/ml TGF-β1 (100-21C, Thermo Fisher, Loughborough, UK) was added to the cells and replenished every other day over a 72-hr time course. Control cells were cultured in low FCS cell culture media and replenished at the same time as those subject to treatment conditions. Cells were then washed twice with 2ml ice-cold phosphate buffered saline (PBS). 500μL of 1X radioimmunoprecipitation assay (RIPA) (20-188, Merk, Gillingham, UK) buffer supplemented with a protease inhibitor cocktail (P8340, Sigma-Aldrich/Merk, Gillingham, UK) was added to the flask of cells, and once cells were dislodged, they were gently agitated on a digital three-dimensional rocker (SK-D1807-S, Scliogex, Rocky Hill, USA) in a 1.5ml microcentrifuge tube for 20 min at 4C. They were then pelleted by centrifuging at 16,000 × g (Megafuge Thermo Fisher, 75004230, Loughborough, UK) for 20 min at 4C. The supernatant was transferred into a fresh 1.5ml microcentrifuge tube and protein concentration of the cell lysates was determined by a Pierce bicinchoninic acid protein assay (23225, Thermo Fisher, Loughborough, UK) according to the manufacturer’s protocol. Absorbance was read using a SpectraMax i3x plate reader (Molecular Devices, Wokingham, UK) at 562nm.

A final concentration of 20μg/μL of protein from the cell lysates was reduced in 0.1M dithiothreitol (R0861, Thermo Fisher, Loughborough, UK) in 4x Laemmli sample buffer (161-0747, Bio-Rad, Watford, UK) at 95C for 5 min. 20μg of samples were then loaded into 8–16% Mini-PROTEAN TGX Stain-Free precast gels (4568103, Bio-Rad, Watford, UK) alongside 5μL of Precision Plus Protein Dual Colour Standards (1610374, Bio-Rad, Watford, UK) and subjected to sodium dodecyl sulphate polyacrylamide gel electrophoresis (SDS-PAGE) in a Bio-Rad Mini-PROTEAN Tetra System for 10 min at 50 volts (V) before increasing to 100V for an additional 1 hr. Proteins were then transferred to low fluorescence 0.45μm pore polyvinylidene difluoride (PVDF) membrane (1704274, Bio-Rad, Watford, UK) using a semi-dry Trans-Blot Turbo transfer system, Trans-Blot Turbo mini-size transfer stacks (1704274, Bio-Rad) in Trans-Blot Turbo 1X transfer buffer (200ml 5X transfer buffer (1704274, Bio-Rad, Watford, UK), 600ml distilled H2O, and 200ml ethanol) . The proteins separated on the gel were transferred onto the membrane using the ‘mixed molecular weight protocol’ setting (2.5 amperes (A), up to 25 V for 7 min).

Membranes were then cut as required and unsaturated protein-binding capacity was blocked in 5% w/v skimmed milk powder (Marvel, Premier Foods, Dublin, Ireland) in Tris buffered saline, pH 7.6 with 1% v/v Tween 20 (P1379, Sigma-Aldrich/Merck, Gillingham, UK) (TBST) for 2hr at room temperature. Membranes were then incubated with primary antibodies for 24 hr at 4C. This included: mouse monoclonal anti-β-actin diluted 1:10,000 (66009-1, Proteintech, Manchester, UK), mouse monoclonal anti-E-cadherin diluted 1:1000 (324102, Biolegend), mouse monoclonal anti-vimentin diluted 1:2000 (677802, Biolegend, San Diego, USA) and mouse monoclonal anti-N-cadherin diluted 1:500 (350802, Biolegend, San Diego, USA), all in 5% w/v skimmed milk powder in TBST. Membranes were then washed three times for 5 min in TBST and then incubated with secondary antibody, goat polyclonal anti-mouse IgG horseradish peroxidase (HRP)-conjugate (W4028, Promega, Southampton, UK) at a dilution of 1:10,000 for vimentin and β-actin labelling and 1:5000 for E-cadherin and N-cadherin labelling, in 5% w/v skimmed milk powder in TBST, for 1 hr at room temperature. Membranes were then washed three times for 5 min each in TBST and then incubated with Clarity western enhanced chemiluminescence (ECL) substrate (170-5061, Bio-Rad, Watford, UK) and imaged using a ChemiDoc imaging system (Bio-Rad, Watford, UK). Band intensity was quantified using Bio-Rad Image Lab software (version 6.1, Bio-Rad, Watford, UK) and was normalised to β-actin labelling, three biological replicates were conducted.

### E-Cadherin, N-Cadherin and Vimentin Immunofluorescence of Cells Following TGF-β1 Treatment

Cells were cultured on coverslips and treated with TGF-β1 as described previously. Cell culture medium was aspirated, and cells were washed with PBS three times. They were then fixed using 4% v/v paraformaldehyde (PFA) (P6148, Sigma-Aldrich/Merck, Gillingham, UK)/PBS for 20 min at 4C. Cells were then washed a further three times with PBS. Cells were then permeabilized using 0.1% v/v Triton X-100 (Sigma-Aldrich/Merck, Gillingham, UK)/PBS for 10 min and then washed a further three times with PBS. They were then incubated for 1 hr at room temperature using 3% w/v bovine serum albumen (BSA)/PBS to prevent non-specific antibody binding. Following this, they were then washed three times with PBS and then incubated with the primary antibody for 1 hr at room temperature; either mouse monoclonal anti-E-cadherin diluted 1:100, mouse monoclonal anti-vimentin diluted 1:500 or mouse monoclonal anti-N-cadherin diluted 1:200, all in 3% w/v BSA/PBS. Primary antibodies were aspirated, and cells were washed 3 times with PBS. Then, a secondary antibody, Alexa Fluor 594-conjugated goat polyclonal anti-mouse IgG (A11005, Thermo Fisher, Loughborough, UK) diluted 1:400 in 3% w/v BSA/PBS, was added and incubated for 1 hr at room temperature. This was then aspirated, and cells were washed three times with PBS. Coverslips were then mounted onto glass slides using ProLong Gold antifade mountant with 4′,6-diamidino-2-phenylindole (DAPI) (P36931, Thermo Fisher, Loughborough, UK) and cured for 24 hr at room temperature in the dark.

**Figure A3.**
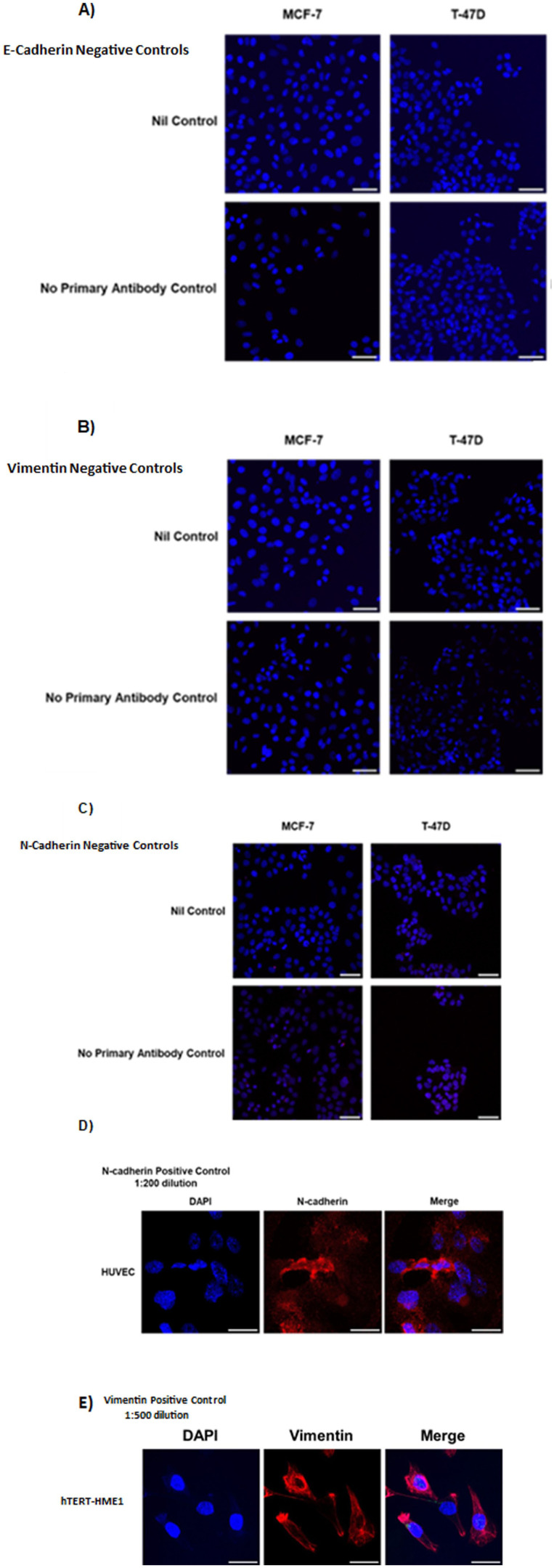
E-cadherin, vimentin and N-cadherin immunofluorescence. Representative Max Intensity Z-stack projections of merged channel. (A) E-cadherin immunofluorescence nil control and no primary antibody control, MCF-7 and T-47D cell lines. E-cadherin (red) and DAPI (blue). Scale bar = 50µm. (B) Vimentin immunofluorescence nil control and no primary antibody control, MCF-7 and T-47D cell lines. Vimentin (red) and DAPI (blue). Scale bar = 50µm. (C) N-cadherin immunofluorescence nil control and no primary antibody control, MCF-7 and T-47D cell lines. N-cadherin (red) and DAPI (blue). Scale bar = 50µm. (D) HUVEC cell line as a positive control for N-cadherin labelling at optimised 1:200 dilution of primary anti-N-cadherin antibody. N-cadherin (red) and DAPI (blue). Scale bar = 25µm. (E) hTERT-HME1 cell line as a positive control for vimentin labelling at optimised 1:500 dilution of primary anti-vimentin antibody. Vimentin (red) and DAPI (blue). Scale bar = 25µm.

Cells were imaged using an upright Zeiss confocal laser scanning microscope (LSM) 800 (Carl Zeiss, Oberkochen, Germany) with a plan-apochromat 63x/1.4 oil DIC objective. Excitation and emission wavelengths were 280nm and 618nm for Alexa Fluor 594-conjugated goat polyclonal anti-mouse IgG (0.2% laser power) and 353nm and 465nm for DAPI (2% laser power). Three images were captured per coverslip, each technical replicate consisted of three coverslips and three biological replicates were conducted. See Appendix Fig. A3 for E-cadherin, vimentin and N-cadherin positive and negative immunofluorescence controls.

Images were quantified in FIJI by creating a sum slice Z-stack projection and the three channels split. Selecting only the DAPI channel, the brightness of the image was elevated until the outline of the cells was visible. The freehand selection tool in FIJI was used to identify cells from the background, and an ROI was created per cell in the image and then added to the ROI manager. Then, the unaltered Alexa Fluor 594-conjugated goat polyclonal anti-mouse IgG antibody channel (EMT marker channel) was selected, ROIs were transposed, and the integrated density of each ROI was measured to determine EMT marker immunolabelling.

### HPA Labelling to Assess Levels of Synthesis of Tn Antigen Following TGF-β Treatment

Cells were cultured on coverslips and treated with TGF-β1 as described previously. Cell culture medium was aspirated from the coverslips and cells were washed three times with PBS. Cells were then incubated for 5 min at 37C with CellMask deep red diluted 1:1000 in PBS (C10046, Thermo Fisher, Loughborough, UK) diluted 1:1000 in PBS, to label the cell membrane, and incubated with 7.5μg/ml Alexa Fluor 488 HPA (Invitrogen / Thermo Fisher, Loughborough, UK) in 3% w/v BSA/PBS for 30 min at room temperature. This was then aspirated, and coverslips were washed three times with PBS. Cells were then fixed using 4% v/v PFA/PBS for 20 min at 4C. Cells were then washed a further three times with PBS. Coverslips were then mounted onto glass slides using ProLong Gold antifade mountant with DAPI and cured for 24 hr at room temperature in the dark.

Cells were imaged using an upright Zeiss confocal laser scanning microscope (LSM) 800 (Carl Zeiss, Oberkochen, Germany) with a plan-apochromat 63x/1.4 oil DIC objective. Excitation and emission wavelengths were 493nm and 517nm for Alexa Fluor 488 HPA (0.2% laser power), 659nm and 676nm for CellMask deep red (0.1% laser power) and 353nm and 465nm for DAPI (1% laser power). Z-stacks of five positions randomly allocated, working outwards from an initial image in an anticlockwise spiral, on the slide were acquired for each coverslip. Each technical replicate consisted of three coverslips and a total of three biological replicates were conducted.

Images were quantified in FIJI; a sum slice Z-stack projection was created and the three channels split. Using the CellMask deep red channel a MinError(I) threshold was applied, and a watershed was applied to identify individual cells creating ROIs. The ROIs were added to the ROI manager alongside one manually drawn ROI background measurement per cell. Corrected total cell fluorescence (CTCF) (= integrated density – (area × mean fluorescence of background measurement)) was calculated based on these measurements.

## Results

### The Effect of TGF-β1 Treatment on MCF-7 and T-47D Cell Morphology

**Figure 2.**
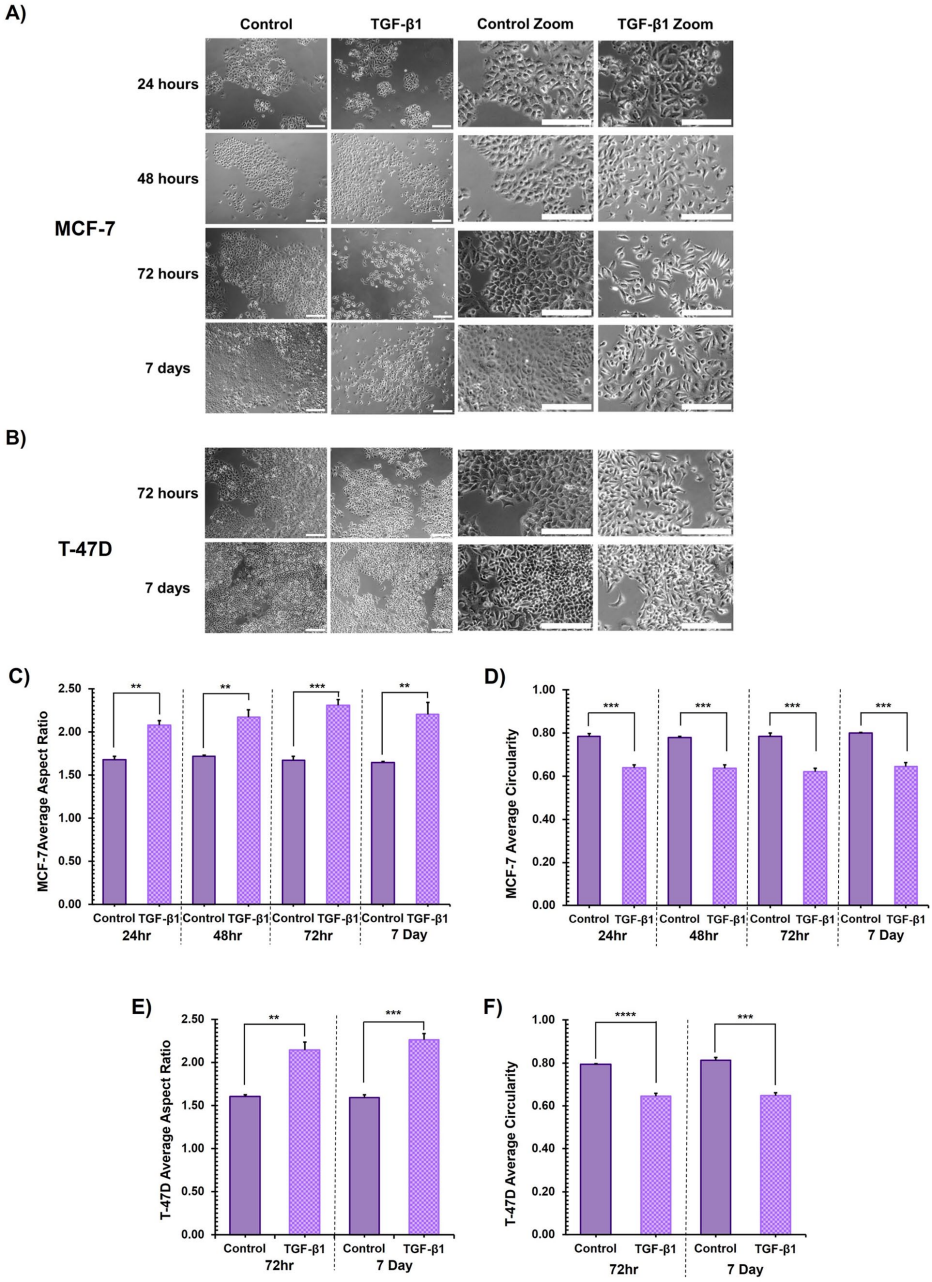
The effect of TGF-β1 treatment on cell morphology. **Upper panel:** Representative phase contrast images of control (untreated) and TGF-β1 treated (A) MCF-7 cells at 24, 48, 72 hr and 7 days and (B) T-47D cells at 72 hr and 7 days. Scale bar = 50μm. **Lower panel:** Morphological assessment of the effect of TGF-β1 treatment on MCF-7 and T-47D cells. (C) Average aspect ratio (±SD) in MCF-7 cells (D) Average circularity (±SD) in MCF-7 cells (E) Average aspect ratio (±SD) in T-47D cells (F) Average circularity (±SD) in T-47D cells. Each biological replicate value consists of an average of three images, and up to 300 cells per image were quantified, three biological replicates were performed. Unpaired Students *t*-test for significance, *p≤0.05, **p≤0.01, ***p≤0.001, ****p≤0.0001.

At all timepoints following 24 hr of TGF-β1 treatment, MCF-7 cells acquired a visually discernible elongated, mesenchymal-like morphology. Control cells retained their cobblestone-like, rounded epithelial morphology throughout all timepoints investigated (Fig. 2A). Assessment of aspect ratio confirmed that TGF-β1 treated MCF-7 cells were significantly more elongated than untreated control cells across all timepoints and consistently demonstrated an aspect ratio score of >2:1, indicating an elongated morphology (Fig. 2C). Analysis of circularity revealed a significantly more rounded morphology in untreated control cells in comparison to TGF-β1 treated cells throughout the time course (Fig. 2D).

The changes in the morphology of T-47D cells were less visibly obvious than those of the MCF-7 cells. However, TGF-β1 treated T-47D cells became visibly more elongated than untreated controls following prolonged exposure, at the later, 72-hr and 7-day, timepoints (Fig. 2B). The aspect ratio of TGF-β1 treated T-47D cells was >2:1, and they were significantly more elongated than untreated control cells (Fig. 2E), indicating an elongated phenotype. Analysis of circularity confirmed that untreated control cells were significantly more rounded than TGF-β1 treated T-47D cells after prolonged exposure (Fig. 2F).

### The Effect of TGF-β1 Treatment on EMT Marker Protein Expression

**Figure 3.**
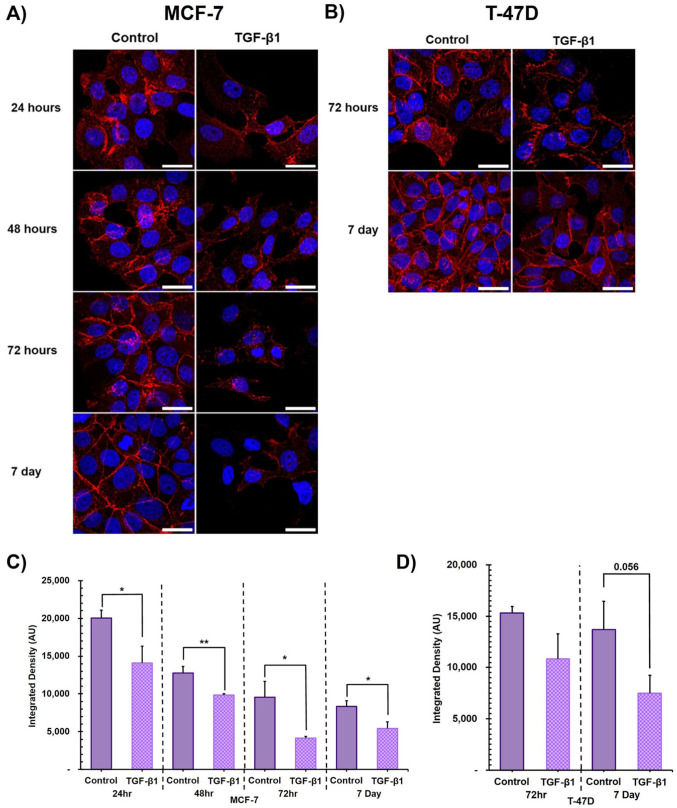
The effect of TGF-β1 treatment on E-cadherin immunofluorescence labelling. **Upper panel:** Representative max intensity Z-stack projections of E-cadherin immunofluorescence of untreated control and TGF-β1 treated (A) MCF-7 and (B) T-47D cells. E-cadherin (red) and DAPI nuclear stain (blue). Scale bar = 25μm. **Lower panel:** Quantification of E-cadherin immunofluorescence of untreated control and TGF-β1 treated cells. Average integrated density (AU) of E-cadherin labelling per cell of (C) MCF-7 and (D) T-47D cells. Three biological replicates (±SD). Unpaired Students *t*-test for significance, *p≤0.05, **p≤0.01.

**Figure A4.**
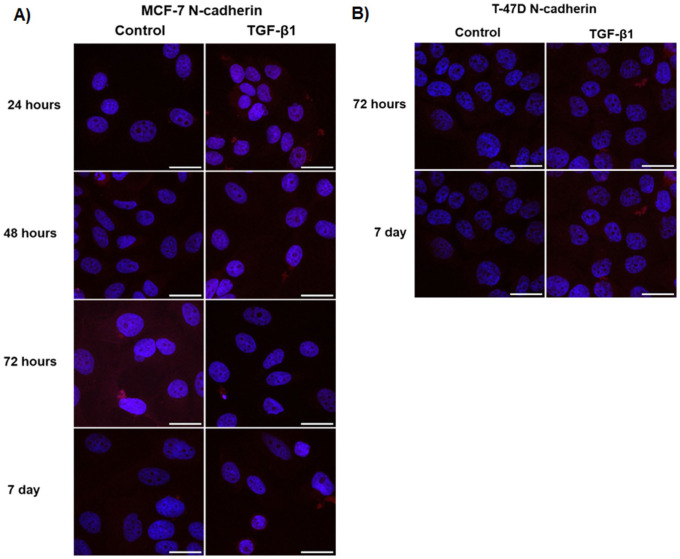
TGF-β1 treatment and N-cadherin immunofluorescence. Representative max intensity Z-stack projections of N-cadherin immunofluorescence in (A) MCF-7, (B) T-47D cell line. N-cadherin (red) and DAPI (blue). Scale bar = 25μm.

**Figure A5.**
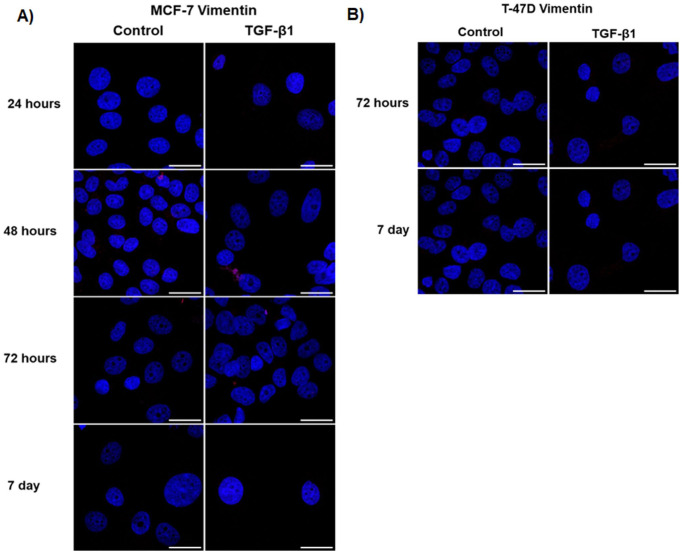
TGF-β1 treatment and vimentin immunofluorescence. Representative max intensity Z-stack projections of vimentin immunofluorescence in (A) MCF-7, (B) T-47D cell line. Vimentin (red) and DAPI (blue). Scale bar = 25μm.

Strong immunolabelling for E-cadherin was apparent in both MCF-7 and T-47D cell line untreated controls, consistent with their epithelial morphology, and across all timepoints investigated (Fig. 3A and 3B respectively). TGF-β1 treated MCF-7 cells demonstrated visibly (Fig. 3A) and quantifiably significantly (Fig. 3C) lower E-cadherin levels than untreated control, across all timepoints, consistent with the morphological changes reported above. TGF-β1 treated T-47D cells demonstrated a visible reduction in E-cadherin protein labelling at both 72 hr and 7 days (Fig. 3B), but, when quantified, this difference did not reach statistical significance (p=0.056 at 7 days, Fig. 3D). Immunolabelling for both N-cadherin and vimentin was negligible and remained unaffected by TGF-β1 treatment over the entire time course in both MCF-7 or T-47D cell lines (see Appendix Figs. A4 and A5).

**Figure 4.**
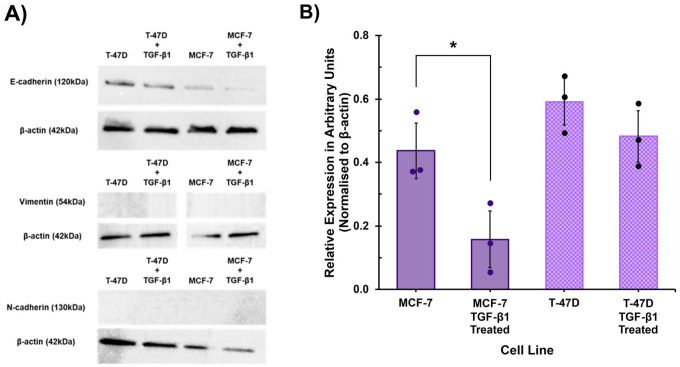
The effect of TGF-β1 treatment on EMT marker protein expression assessed by western blot. (A) Representative western blots for E-cadherin (120kDa), vimentin (54kDa) and N-cadherin (130kDa) and loading control β-actin (42kDa) for control (untreated) and TGF-β1-treated T-47D and MCF-7 cells at 72 hr. (B) Relative protein levels of EMT marker E-cadherin normalised to β-actin in control (untreated) and TGF-β1 treated MCF-7 and T-47D cells, displayed as average of three biological replicates (±SD). Unpaired Students *t*-test for significance, *p≤0.05.

**Figure A6.**
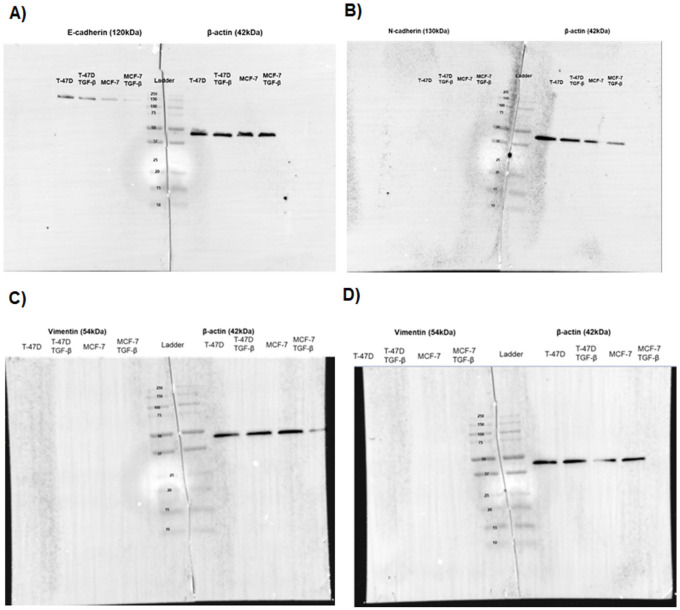
Original western blots TGF-β1 treatment and control conditions for (A) E-cadherin (120kDa) for both MCF-7 and T-47D cells, (B) N-cadherin (130kDa) for both MCF-7 and T-47D cells, (C) and (D) repeat blots of vimentin (54kDa) for both MCF-7 and T-47D cells.

Immunofluorescence to visualise EMT protein marker expression was confirmed by western blot analysis. There was a clear, visibly discernible, reduction in E-cadherin protein levels for both MCF-7 and T-47D cell lines treated with TGF-β1 compared to untreated control at the 72-hr timepoint (Fig. 4A). Relative protein levels of E-cadherin, normalised to β-actin, in TGF-β1 treated MCF-7 cells was significantly lower compared to untreated control (*p*<0.05) (Fig. 4B). The reduction of E-cadherin in TGF-β1 treated T-47D cells compared to untreated control did not reach statistical significance (Fig. 4B), consistent with the results of the immunofluorescence labelling. Neither N-cadherin or vimentin proteins were detectable in either the untreated control or TGF-β1 treated cells by western blot (Fig. 4A, and see Appendix Fig. A6), again consistent with the immunofluorescence labelling results.

### The Effect of TGF-β1 Treatment on Levels of Synthesis of Tn Antigen, Detected by HPA Labelling

**Figure 5.**
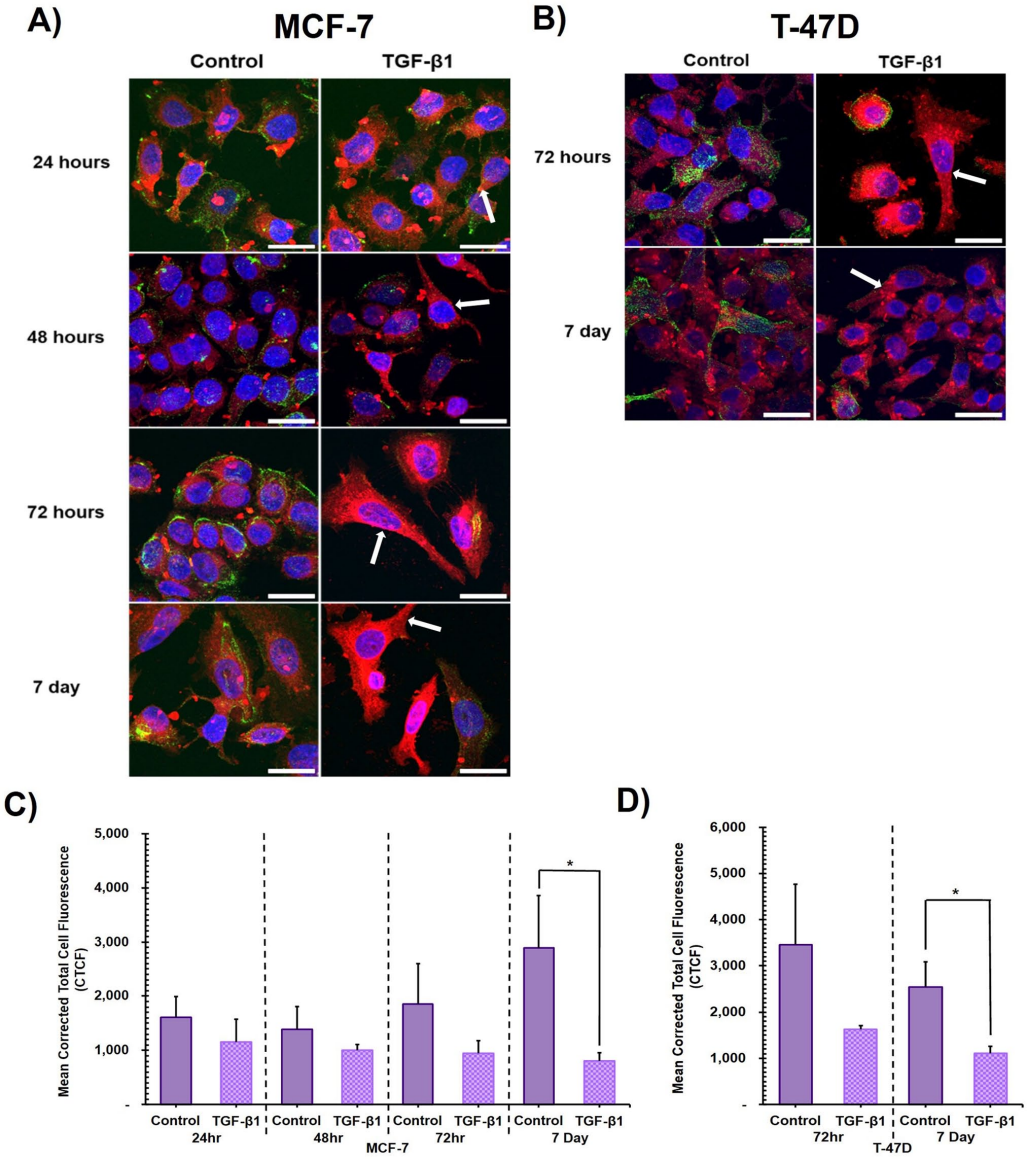
The effect of TGF-β1 treatment on levels of synthesis of unelaborated O-GalNAc glycans, detected by HPA labelling. **Upper panel:** Representative max intensity Z-stack projections of fluorescent HPA lectin labelling in (A) MCF-7 and (B) T-47D cells over a time course. HPA (green) for O-GalNAc glycans, CellMask for plasma membrane labelling (red) and DAPI nuclear stain (blue). Arrows highlight examples of cells exhibiting negligible HPA lectin labelling and elongated morphology following TGF-β1 treatment. Scale bar = 25μm. **Lower panel:** Mean corrected total cell fluorescence (CTCF) for HPA labelling in TGF-β1-treated cells over a time course. CTCF of HPA labelling in (C) MCF-7 untreated control and TGF-β1 treated cells, (D) T-47D untreated control and TGF-β1 treated cells. Three biological replicates (±SD). Unpaired Students *t*-test for significance, *p≤0.05.

TGF-β1 treatment of both MCF-7 and T-47D cells visibly impacted the levels of cell surface Tn antigen, detected by HPA labelling. At all timepoints, 24, 48, 72 hr and 7 days, MCF-7 cells showed progressively visibly reduced O-GalNAc glycan labelling compared to untreated controls. Corresponding changes in cell morphology, to an elongated mesenchymal-like morphology, were also readily visibly apparent in HPA-negative cells (Fig. 5A, examples indicated by arrows). The observed reduction in HPA-positivity, when quantified, reached statistical significance by 7 days (Fig. 5C). It was striking that the entire cell population did not become HPA-negative upon TGF-β1 cytokine treatment, with a small population of cells, or individual cells, retaining cell surface HPA-binding O-GalNAc glycans over the entire time course.

At timepoints 72 hr and 7 days TGF-β1 treated T-47D cells also exhibited noticeably visibly reduced levels of O-GalNAc glycans compared to untreated control. These changes were also associated with a morphological change to a mesenchymal-like, elongated phenotype (Fig. 5B, examples indicated by arrows). The observed reduction in HPA-positivity reached statistical significance by 7 days, compared to untreated control (Fig. 5D).

## Discussion

This study examined the effect of TGF-β1 cytokine treatment on O-GalNAc glycosylation and EMT in MCF-7 and T-47D breast cancer cells. TGF-β1 treatment induced changes consistent with induction of EMT in both cell lines; they acquired a more mesenchymal morphology following TGF-β1 treatment, across each timepoint investigated up to 7 days of cytokine treatment. In both cell lines, TGF-β1 treated cells became significantly more elongated, breaching the >2:1 aspect ratio, and were less circular than untreated control cells. These morphological changes are consistent with other studies that have also demonstrated that TGF-β1 treatment, at varying concentrations and exposure timepoints, induces morphological changes in these cell lines, MCF-7^26^,^28,29^ and T-47D,^26^ consistent with induction of EMT.

In addition to morphological changes, TGF-β1 cytokine treatment also resulted in the reduction of the key epithelial marker E-cadherin, which has been referred to as the ‘gatekeeper of the epithelial state’,^35^ and an important tumour suppressor gene. Reduced expression or loss of E-cadherin is regarded as the major mechanism of triggering the loss of cell-cell adhesion and, therefore, initiating and promoting cell migration and metastasis.^36^ In MCF-7 cells, there was both a visibly discernible and statistically significant reduction in E-cadherin across all timepoints investigated (24, 48, 72 hr and 7 days), assessed by immunolabelling. In T-47D cells, E-cadherin immunolabelling was decreased visibly at 72 hr, and this reduction was nearing statistical significance (p=0.056) after 7 days of TGF-β1 treatment. However, in both cell lines, there was no concomitant visibly discernible induction of N-cadherin or vimentin protein expression following TGF-β1 cytokine treatment, although positive immunofluorescence of control cells (HUVEC and hTERT-HME1, respectively) confirm that the labelling protocol was effective. Moreover, total RNA sequencing confirmed differential expression of key EMT-inducing transcription factors SNAI1, SNAI2 and SNAI3 in MCF-7 cells in response to TGF-β1 treatment (Appendix Table A1).

Loss of E-cadherin via post-translational downregulation has been described as a marker of ‘early EMT’,^37,38^ whereas N-cadherin expression has been described as a marker of a ‘late EMT’ state.^39^ The results presented here could therefore be indicative of an early or partial EMT state, with cells showing a reduction in the epithelial marker E-cadherin and not having yet gained expression of mesenchymal markers. As Fontana et al.^40^ recently discussed, it is increasingly recognised that intermediate EMT states rather than a binary switch are characteristic of cancers, and such EMT plasticity functionally enhances tumour progression, generation of cancer stem cells and resistance to therapies. It is interesting to note that the TGF-β1 treated MCF-7 and T-47D cells did not fully lose E-cadherin protein expression, despite the large reduction compared to untreated controls, also consistent with a partial EMT state. Aban et al.^38^ demonstrated in human embryonic stem cells that partial downregulation of E-cadherin, achieved by CRISPR, resulted in a partial EMT state whereby cells demonstrated some characteristics of a mesenchymal phenotype. It also stimulated collective cell migration, a characteristic of partial EMT, and increased ‘wound’ closure in a scratch assay. The partial loss of E-cadherin could act as the triggering signal at the start of EMT.

As mentioned previously, studies have reported vimentin and / or N-cadherin expression in the cell lines used in the current study following TGF-β treatment at the protein or mRNA level, as well as, in some instances, evidence of other EMT-related changes such as upregulation of transcription factor mRNA and changes in cell behaviour, such as cell invasiveness or motility.^25–29^ The reasons why, in comparison, only partial EMT was achieved in the current study are not obvious but may be related to the inevitable small differences in cell line phenotypes, conditions under which cytokine stimulation was performed, timepoints assessed, and protocols for western blot and immunocytochemistry. As an example of the variability in reported results between apparently similar studies, results presented here indicate that neither N-cadherin nor vimentin protein were detectable in untreated control cells. This is entirely consistent with several other reports in the literature that neither MCF-7^41^ nor T-47D cells^42,43^ normally express N-cadherin protein, or vimentin protein.^44–46^ However, one study^26^ which reported TGF-β1 treatment to induce morphological changes, downregulation of E-cadherin and upregulation of vimentin in both MCF-7 and T-47D cell lines at protein and mRNA level (as detected by western blotting and qRT-PCR, respectively) also, surprisingly, reported high levels of vimentin protein in untreated cells. It is also possible that in the study reported here, broader exploration of EMT marker expression, or investigation of cell behaviour – for example, through wound healing or Transwell assays – may have revealed other effects consistent with induction of EMT.

Suppression of the epithelial marker E-cadherin and induction of the mesenchymal markers N-cadherin and vimentin are arguably the most recognised signatures of EMT, and this was the reason for their choice as markers of EMT induction in the current study. Moreover, the focus of the current study was on detection of the final protein product expressed by cells, as detected through immunocytochemistry and western blot. A portfolio of other markers could have been explored, including EMT-orchestrating transcription factors, such as the Snail family transcriptional repressors (SNAI1/SNAIL, SNAI2/SLUG, SNAI3), Zinc finger E-box binding homeobox 1 and 2 (ZEB1, ZEB2), Twist family transcription factors (TWIST1, TWIST2), reduction of expression of other epithelial proteins such as zonula occludens protein-1, ZO-1 (TJP1), occludin (OCLN) and desmoplakin (DSP), and promotion of mesenchymal proteins such as fibronectin (FN1) and vitronectin (VTN), and many others.^24,47^ Furthermore, evidence of their induction or repression could have been sought through, for example, assessment of mRNA levels through qPCR or more comprehensive total RNA sequencing. However, since, as described previously, it is well established in the literature that TGF-β induces epithelial cells, including MCF-7 and T-47D breast cancer cell lines, to progress towards EMT,^24–29^ consistent with observations reported here, and since the focus of the study was to explore the effect of such stimulation on glycosylation, further examination of EMT markers was not undertaken.

Notably, in the study reported here, TGF-β1 treatment resulted in a significant reduction in the level of cell surface Tn antigen, detected by HPA binding, which corresponded to changes in cell morphology to a more elongated mesenchymal-like morphology. This, intriguingly, may be indicative of a potential role of altered O-glycosylation in early or partial EMT states. This is the first time that alterations in Tn antigen synthesis have been documented as changing in response to TGF-β1 induced EMT in breast cancer cell lines. In the absence of TGF-β1 treatment, MCF-7 cells frequently demonstrated intense HPA labelling at the cell membrane with, sometimes, highly clustered perinuclear labelling. T-47D cells displayed a slightly different pattern of localisation with a generally more diffuse punctate labelling across the cell surface. In both cases, labelling is consistent with the synthesis and exposure of unelaborated Tn antigen at the cell surface. TGF-β1 treatment induced both MCF-7 and T-47D breast cancer cells to display significantly less Tn antigen at the 7-day timepoint. Interestingly, in neither cell line did the entire cell population become HPA-negative upon TGF-β1 treatment; instead, a small population of cells, or individual cells, retained cell surface Tn antigen. This observation is intriguing since it reflects observations reported in studies of HPA labelling of histological sections of clinical breast cancers where, commonly, a majority – but not all – of cancer cells within a tumour are positive or, conversely, the majority – but not all – are negative.^6,48^ Moreover, we have previously reported on the characterisation of several breast cell lines for their stable levels of synthesis of O-GalNAc glycans detected by HPA, where we routinely see populations of cells within an individual cell line exhibiting very different glycosylation patterns,^49^ suggesting stable and subtle control of glycosylation patterns within cell sub populations even within an otherwise apparently heterogeneous population of cells. Consistent with results presented above, untreated HPA-negative MCF-7 cells separated from a standard cell culture preparation showed differential expression of EMT-related SNAI2/SLUG and ZEB1 RNA, and untreated HPA-negative T-47D cells showed differential expression of ZEB-1, in comparison to untreated HPA-positive cells separated from the same culture (Appendix Table A1). Evidence presented here is consistent with such glycosylation patterns reflecting the state of the cells in relation to their transition to EMT, and plasticity in glycoprofiles.

Much of the literature reporting on the effect of TGF-β1 treatment on glycosylation has focussed on the N-glycome and frequently describes changes in N-linked glycosylation and expression of correlating glycosylation enzymes following TGF-β1 treatment. However, only a handful of studies have specifically explored O-glycosylation of cells after TGF-β1 treatment. As an example, Lucena et al.^50^ reported that treating A459 lung cancer cells with TGF-β1 for up to 48 hr induced EMT, as detected by changes in morphology and altered EMT protein marker expression, reduced E-cadherin and increased vimentin. Using a variety of lectins in flow cytometry, they demonstrated a significant increase in labelling for *Sambucus nigra* agglutinin (SNA) indicating sialyl Tn antigen, and a visible, but non-significant, decrease in peanut agglutinin (PNA) indicating core 1. Consistent with the results reported here, they also saw a decrease in labelling with *Vicia villosa* agglutinin (VVA), which has a similar specificity to HPA for O-GalNAc glycans. Yang et al.^51^ reported how treating bovine chondrocytes with TGF-β led to a reduction in sialyl core 1 glycans and corresponding increase in unelaborated core 1, assessed by *Maackia amurensis* agglutinin (MAA) and peanut agglutinin binding, respectively, in enzyme-linked lectin assays (ELLAs). Xiao et al.^52^ demonstrated an increase in both Tn antigen and core 1 on IgA secreted by human B lymphoma cells following TGF-β stimulation, assessed using *Vicia villosa* agglutinin and peanut agglutinin binding in ELLA. Zhang et al.^53^ demonstrated increased core 1 and decreased core 2, core 4 and sialyl Tn glycans, detected by mass spectrometry, after TGF-β stimulation of human pancreatic ductal adenocarcinoma cells. The cells also showed changes in a range of EMT-related markers and increased invasive capacity in a zebrafish model. Elaboration of Tn antigen to core 1, and/or sialylation of these structures to form sialyl Tn and sialyl core 1, respectively, were not explored in the current study but their enhanced synthesis could provide a potential explanation for the reduction in exposed O-GalNAc, detected by HPA labelling, seen following TGF-β1 treatment and accompanying EMT in both MCF-7 and T-47D cells.

Cumulatively, this evidence suggests that TGF-β1 induces EMT and concomitant changes in O-linked glycosylation, specifically, changes in synthesis and exposure of truncated O-glycans, including Tn antigen. The presence of these truncated O-glycans have repeatedly been described in the literature to be associated with metastasis and poor prognosis in a range of epithelial cancers, including breast cancer. Despite such glycosylation changes being well established, surprisingly little is understood of their functional significance in metastatic mechanisms. We have previously reported evidence that Tn antigen, the presence of which is strongly associated with metastatic potential in breast cancer, is involved in adhesion of breast cancer cells to endothelial monolayers, consistent with it playing a functional role in this stage in hematogenous metastasis.^54^ In contrast, we found no evidence that this metastasis-related glycan was involved in a different stage in the metastatic cascade, adhesion to, or invasion through, basement membrane components.^49^ As described previously, it is increasing accepted that cancer cells commonly exhibit a mixture of epithelial and mesenchymal states, partial EMT, and considerable plasticity in relation to these states. Moreover, during metastasis, maintenance of their phenotype will be dependent on local contextual signals, including the presence of inflammatory cytokines like TGF-β1. Given the reciprocal relationship between EMT and glycosylation, such plasticity may be mirrored in cancer cells exhibiting a flexible portfolio of glycomotifs at different stages in metastasis that, at each stage, have distinct functional significance. A clearer understanding of such mechanisms, and of the involvement of altered glycosylation in cancer progression, is of considerable biological interest, and may ultimately lead to development of novel preventative or therapeutic approaches.

## Appendix

**Table A1.** EMT-Related Transcription Factor DEGs (Differentially Expressed Genes) Up-Regulated by TGF-β1 Treatment, and Associated With Loss of Cell Surface Tn Antigen, Detected by HPA Binding.

Cells	Gene	Transcription factor	Fold-change	Regulation direction	***p***-value
HPA-negative vs HPA-positive MCF-7	SNAI2/SLUG	Snail Family Transcriptional Repressor 2, Snail2, Slug	1.61	Up-regulated	*p*<0.025
	ZEB1	Zinc finger E-box binding homeobox 1	1.45	Up-regulated	*p*<0.025
HPA-negative vs HPA-positive T-47D	ZEB1	Zinc finger E-box binding homeobox 1	1.54	Up-regulated	*p*<0.025
TGF-β1 stimulated MCF-7 vs HPA-positive MCF-7	SNAI1/SNAIL	Snail family transcriptional repressor 1, Snail	1.41	Up-regulated	*p*<0.05
	SNAI2/SLUG	Snail Family Transcriptional Repressor 2, Snail2, Slug	2.44	Up-regulated	*p*<0.025
	SNAI3	Snail family transcriptional repressor 3, Snail3	1.40	Up-regulated	*p*<0.025

Total RNA sequencing was conducted to determine transcriptomic differences between HPA-positive and HPA-negative cells separated from MCF-7 and T-47D cell lines using HPA-coated magnetic beads, and between TGF-β1 treated (72-hr timepoint, 10ng/ml TGF-β1) and HPA-positive MCF-7 cells.
